# Integrated transcriptome and single-cell sequencing analysis identify blood-pancreas shared lncRNA biomarkers in new-onset T2DM

**DOI:** 10.1371/journal.pone.0345359

**Published:** 2026-03-31

**Authors:** Yan Wang, Yangming Qu, Mingxin Dong, Yulong Zhang, Na Zhao, Suli Song, Mingyang Huo, Nan Lu, Baichuan Chen, Chengbiao Sun, Kaikai Yu, Juan Ren, Na Xu, Wensen Liu, Yan Yao

**Affiliations:** 1 Department of Epidemiology and Biostatistics, School of Public Health, Jilin University, Changchun, China; 2 State Key Laboratory of Pathogen and Biosecurity, Changchun Veterinary Research Institute, Chinese Academy of Agricultural Science, Changchun, China; 3 Department of Clinical Laboratory, Xing'an League People's Hospital, Ulanhot, China; 4 Jilin Medical University, Jilin, China; 5 Weifang People's Hospital, Weifang, China; Aarupadai Veedu Medical College & Hospital, INDIA

## Abstract

Type 2 diabetes mellitus (T2DM) is characterized by β-cell dysfunction and insulin resistance, yet the early molecular drivers remain elusive. This study set out with the aim of identifying blood-pancreas shared long non-coding RNAs (lncRNAs) as potential systemic biomarkers in treatment-naïve patients with new-onset T2DM. We integrated transcriptome sequencing of peripheral blood from 8 T2DM patients and 8 controls with single-cell RNA sequencing (scRNA-seq) of pancreatic islets from an independent cohort. Differential expression analysis revealed 1,709 dysregulated lncRNAs in peripheral blood, of which 257 were identified as high-priority candidate through weighted gene co-expression network analysis (WGCNA). Further intersection with scRNA-seq data from 17 T2DM donors identified 157 β-cell-specific mRNAs co-expressed with 135 blood-derived lncRNAs. Functional enrichment analysis implicated these genes in chromatin remodeling, focal adhesion, and neurodegenerative pathways. Validation in an expanded cohort (85 T2DM vs. 85 controls) confirmed significant downregulation of ENST00000473095, MSTRG.90147.1 and ENST00000531992 in T2DM. The combined ROC-AUC value of these three lncRNAs was 0.73, which exceeds the AUCs of each individual lncRNA (0.61–0.64). Our findings tentatively suggest that blood-derived lncRNAs as early biomarkers reflecting β-cell stress and systemic dysregulation. These lncRNAs may potentially bridge peripheral blood biomarkers with tissue-specific pathophysiology in T2DM.

## Introduction

Type 2 diabetes mellitus (T2DM) represents a significant global health challenge, impacting more than 537 million adults worldwide, with projections indicating an alarming increase to approximately 783 million cases by 2045 [1]. The diabetes epidemic places an immense strain on healthcare systems because of the severe complications caused by chronic hyperglycemia. This underscores an urgent need for strategies aimed at early detection and intervention. The disease is characterized by progressive β-cell dysfunction and insulin resistance, which together result in hyperglycemia and a cascade of systemic complications that can severely affect an individual's quality of life. Despite notable advances in our understanding of its complex pathophysiology, the intricate molecular mechanisms that underlie early-stage T2DM remain inadequately elucidated, particularly within pancreatic tissue, which is crucial in the pathogenesis of this debilitating disease [2]. A significant limitation in the field is the majority of existing studies have concentrated on established T2DM or medicated cohorts, thereby introducing potential confounding factors stemming from chronic hyperglycemia or the effects of pharmacotherapy, which may obscure the true nature of the disease's onset. Investigating newly diagnosed, treatment-naïve individuals provide a unique and invaluable opportunity to identify the early molecular drivers of T2DM, potentially facilitating timely and targeted interventions that could effectively halt or even reverse disease progression before it leads to more severe health consequences.

Long non-coding RNAs (lncRNAs), which were once dismissed as mere transcriptional “noise” in the complex landscape of gene expression, have now emerged as pivotal regulators of metabolic homeostasis, fundamentally altering our understanding of cellular biology. Recent studies have illuminated their significant and nuanced roles that are specific to various tissues, particularly highlighting their importance in the survival of pancreatic β-cells and in the broader context of systemic insulin sensitivity [3,4]. Unlike traditional protein-coding genes, lncRNAs display distinct expression patterns that vary from one tissue to another, positioning them as highly promising candidates for the development of disease-specific biomarkers that could revolutionize diagnostic approaches. For instance, LINK-A, a lncRNA that facilitates the linking of kinase activation, has been demonstrated to directly interact with AKT/PKB signaling pathway within human islets, thereby enhancing glucose-dependent insulin signaling cascades in a manner that is intricately tied to glucose levels [5]. Likewise, cutting-edge single-cell RNA sequencing of pancreatic islets derived from donors with T2DM has identified βLinc1 as a crucial lncRNA that plays a vital role in preserving β-cell identity by stabilizing the transcriptional networks governed by PDX1 [6]. Despite these significant advancements in our understanding of lncRNAs, current research efforts are largely hampered by a reliance on peripheral blood or surrogate tissues, primarily due to the challenges associated with obtaining human pancreatic biopsies. This constraint obscures our ability to fully elucidate the pancreas-specific lncRNA-mRNA interactions that are essential for unraveling the early pathogenesis of T2DM, thereby highlighting a critical gap in our knowledge that warrants further investigation.

Recent advances in the field of single-cell RNA sequencing (scRNA-seq) have significantly transformed our understanding of the intricate cellular heterogeneity present within pancreatic islets. This cutting-edge technology has unveiled distinct subpopulations of β-cell, each exhibiting varying degrees of susceptibility to metabolic stress [7–9], thereby highlighting the complexity of cellular responses in this critical tissue. However, a major challenge persists in the form of the inaccessibility of human pancreatic tissue, which routinely limits the clinical translation of these groundbreaking findings into practical applications. Conversely, peripheral blood offers a readily accessible source for potential biomarkers, however, it often falls short in providing direct mechanistic links to the underlying pathophysiology of the tissue. The integration of bulk transcriptome data from blood samples with single-cell resolution analyses of pancreatic tissue emerges as a powerful and promising strategy. This innovative approach holds the potential to identify lncRNAs that demonstrate coordinated expression patterns in both whole blood and pancreatic tissue, thereby bridging the gap between easily obtainable blood biomarkers and the complex dynamics of pancreatic function. Such molecules could serve dual roles, functioning not only as systemic biomarkers but also offering direct insights into β-cell dysfunction [10]. Despite the considerable potential of these integrative analytical approaches, they remain notably underutilized in studies focused on new-onset T2DM. The detection of early molecular signatures through such methodologies could yield the most clinically actionable insights, ultimately enhancing our ability to intervene in this prevalent and challenging metabolic disorder.

This study, therefore, aimed to identify novel blood-pancreas shared lncRNA biomarkers in treatment-naïve, new-onset T2DM by integrating comprehensive bulk transcriptomic profiling of peripheral blood with advanced scRNA-seq of human pancreatic islet. We hypothesized that such an integrative multi-omics approach would uncover blood-derived dysregulated lncRNAs that reflect early β-cell stress and systemic metabolic dysfunction, potentially offering superior insights into early disease mechanisms compared to conventional clinical metrics that often fall short in capturing the complexity of the disease. Ultimately, our findings seek to bridge the gap between accessible peripheral blood biomarkers and the intricate tissue-specific pathophysiology of T2DM, paving the way for innovative diagnostic strategies that could significantly enhance early detection and intervention in this prevalent metabolic disorder.

## Materials and methods

### Patients’ enrollment

In this research, a total of eight individuals diagnosed with first-onset type 2 diabetes mellitus (T2DM) were enrolled, alongside eight healthy controls (CTL) matched for age and gender, forming a cohort for high-throughput sequencing analysis. The diagnostic criteria for T2DM were based on the 1999 WHO Diagnostic Standard [11]. Specifically, they were confirmed with 1) a fasting blood glucose (FBG) ≥ 7.0 mmol/L, oral glucose tolerance test (OGTT) two-hour blood glucose ≥ 11.1 mmol/L, or random blood glucose ≥ 11.1 mmol/L; 2) an HbA1c% level ≥ 6.5%; 3) simple T2DM excluding other complications such as hypertension, coronary heart disease, stroke, tumor, acute infectious disease, immune and hematological disease, hepatitis, etc.; and 4) no history of medication for T2DM and the above-mentioned diseases. The 8 healthy control subjects were physically normal individuals with no history of medication use for T2DM and related diseases during the same period. To ensure the statistical power of the validation, the minimum validation sample size, 48 cases and 48 controls, was calculated based on the pre-experiments of 10 cases and 10 controls using GPower 3.1.9.7, while the effect size d was 0.75 with a power of 95%. Finally, the validation cohort consists of 85 T2DM patients and 85 CTLs. The inclusion and exclusion criteria are the same as the sequencing cohort. All subjects were recruited from 01 August 2023–31 December 2024.

### Ethical approval statement

This study was performed in line with the principles of the Declaration of Helsinki and was approved by the Medical Ethics Committee of Xing’ an League People's Hospital, and written informed consent was obtained from all participants.

### Blood sampling and total RNA extraction procedure

Whole blood (3 mL) from all subjects was collected in the early morning after overnight fasting in polypropylene tubes containing EDTA anticoagulant, processed with 9 mL of TRIzol reagent (Sangon, China), and stored at −80°C until RNA extraction. When total RNA was extracted, the mixed solution was transferred to RNase/DNase-free EP tubes, chloroform was added at a ratio of 1:0.2 and shaken well, and after standing at room temperature for 5 min, it was centrifuged at 8,000 rpm at 4℃ for 25 min. Then, the supernatant was collected, and 1/2 volume of anhydrous ethanol was added to the supernatant and mixed well. Then, the RNA was purified according to the protocol of the RNA-Quick purification kit manufacturer (ES Science, China). After washing and centrifugation of the total RNA pellet, the total RNA was dissolved in 30 μL elution buffer and stored at −80℃ until analysis. The Eva3200 Ultra Trace Nucleic Acid Protein Detector (Monad, China) was used to check the purity and concentration of the RNA, and the Agilent Bioanalyzer 2100 system (Agilent Technologies, USA) was used to assess the integrity of the RNA.

### RNA extraction library construction and sequencing

8 T2DM patients and 8 CTLs were recruited, and high-throughput sequencing of their peripheral blood RNA was performed. Briefly, Total RNA was isolated from samples using Trizol reagent (Thermofisher, USA) following standard protocols. RNA quality assessment was performed using an Agilent Bioanalyzer 2100 with RNA 6000 Nano LabChip Kit (Agilent, USA), maintaining RNA integrity numbers (RIN) >7.0. Ribosomal RNA depletion was conducted using 5 μg total RNA with the Ribo-Zero Gold rRNA Removal Kit (Illumina, USA). Subsequent RNA fragmentation (NEB，USA) generated 200–300nt fragments through magnesium-catalyzed hydrolysis at 94°C. First-strand cDNA synthesis was performed using SuperScript™ II Reverse Transcriptase (Thermofisher, USA), followed by second-strand synthesis with dUTP incorporation via E. coli DNA polymerase I and RNase H (NEB，USA). Following end-repair and A-tailing, Illumina adapters with unique indexes were ligated to 300–600 bp size-selected fragments using AMPureXP beads. Uracil excision was implemented through UDG treatment (NEB，USA) prior to PCR amplification (95°C/3 min; 8 cycles of 98°C/15s, 60°C/15s, 72°C/30s; final extension 72°C/5 min). Final cDNA libraries showed average insert sizes of 300 ± 50 bp. Paired-end sequencing (2 × 150 bp) was executed on an Illumina Novaseq™ 6000 platform (LC-Bio Technology, China) following manufacturer specifications. This optimized workflow ensured high-quality strand-specific transcriptome data acquisition while maintaining compatibility with downstream bioinformatics analyses.

### Analysis of differential expression lncRNAs (DE lncRNAs)

Three methods, limma, DESeq2 and edgeR, were used to identify DE lncRNAs between T2DM patients and healthy controls from bulk RNA sequencing data, and values of |log_2_FoldChanges| ≥ 1 and *p* < 0.05 were set as thresholds to screen out significant DE lncRNAs. To evaluate the reliability of the differential expression analyses, we performed Spearman correlation analysis to assess the pairwise correlation of log_2_FoldChanges obtained from the three R packages. We intersected the results of the three sets of DE lncRNAs to screen out solid DE lncRNAs of T2DM. Based on transcriptomic profiling data, we performed comprehensive visual analysis of differential gene expression patterns utilizing specialized bioinformatics tools in R environment (version 4.2.1). The analytical workflow incorporated established visualization packages including ‘pheatmap’ (v1.0.12) for hierarchical clustering heatmaps, ‘ggplot2’ (v3.4.0) for multivariate data visualization, and ‘UpSetR’ (v1.4.0) for set relationship analysis, following established computational biology protocols.

### Weighted gene Co-expression network analysis (WGCNA)

We used the isoforms expression profiles from the RNA sequencing data to construct the co-expression network using the R package “WGCNA”. The adjacency matrix was converted into a topological overlap matrix (TOM). A TOM heatmap was constructed using the “Tomplot” function in the “WGCNA” R package. Subsequently, we screened out the power parameter ranging from 1 to 20 using the “pickSoftThreshold” function in the “WGCNA” package, then genes were divided into different modules. Finally, T2DM-related modules were obtained, and the resulting gene network was visualized using a heatmap.

### Target gene prediction of lncRNAs

To explore the function of lncRNAs, we combined the results of standardized difference analysis and WGCNA to predict cis- and trans-target genes of differentially expressed lncRNAs. In this study, cis-prediction was performed on coding genes in 100,000 bp upstream and downstream using a Python script. Trans-prediction was performed using the blat tool based on the correlation coefficient between lncRNA and mRNA expression (correlation coefficient corr ≥ 0.9).

### Acquisition and processing of scRNA-seq data

The single-cell RNA sequencing (scRNA-seq) dataset GSE221156, comprising 17 T2DM samples and 17 controls, was procured from the Gene Expression Omnibus (GEO) database. The scRNA-seq data analysis was conducted utilizing the Seurat 4.0 package in R. Briefly, a Seurat object was created by importing the sample expression matrices into R via the Read10 × function, alongside the integration of pertinent clinical information. The quality control process involved several steps. Firstly, only cells with gene expression counts between 300 and 1,000 were retained. Additionally, cells with more than 10% of their reads mapped to the mitochondrial genome, indicative of poor cell quality, were excluded. Doublets, or cells erroneously identified as single cells, were also removed from each sample to ensure dataset purity and accuracy. Following quality control, highly variable genes (HVGs), which are crucial for distinguishing between different cell types, were identified. The top 2000 HVGs were selected for further analysis. Using these genes, Principal Component Analysis (PCA) was performed to reduce the dataset's dimensionality and identify the main axes of variation. Based on the PCA results, cluster analysis was conducted using the top 1–41 principal components. To explore the role of islet cells in T2DM, a set of differentially expressed genes among all islet cell subsets was obtained. Briefly, the Findallmarkers function was employed to identify islet cell subsets and calculate the DEGs from each cell subpopulation, with a threshold of *p*  <  0.05. This gene set, subsequently used for consensus clustering, encompassed all potential characteristic genes of the islet cell subpopulation.

### Functional enrichment analyses

To obtain the biological functions and signaling pathways of the genes, we used the R package clusterProfiler for gene ontology (GO) annotation and Kyoto encyclopedia for genes and genomes (KEGG) pathway enrichment analysis, respectively. *p* < 0.05 was set as the threshold for the identification of related GO functions and KEGG pathways, and the top 10 results were visualized in the form of bubble charts.

### Peripheral blood lncRNA validation by qRT-PCR

Validation analysis was performed in an independent cohort of 170 individuals (85 T2DM vs. 85 CTLs) using real-time PCR. Specific PCR primers for the ten candidate biomarkers were synthesized by Sangon Biotech (Sangon Biotech, China). Primer sequences are listed in S1 Table. Rapid reverse transcription of RNA into cDNA was performed following the protocols of the reverse transcription kit (Thermo Fisher, USA). The total PCR reaction volume was 10 μL, consisting of 5 μL Power Up ^TM^ SYBR Green Master Mix, 0.4 μL forward primer, 0.4 μL reverse primer, 2 μL cDNA, and 2.2 μL free water. The reaction condition was 95℃ for 10 min and 40 cycles of 95℃ for 15 s, 60℃ for 1 min, and 95℃ for 15 s. Finally, data were collected for statistical analysis. GAPDH was used as an internal reference gene to normalize lncRNA expression levels. The relative expression level of lncRNAs was calculated by the 2^-△△Ct^ method. All reactions were performed in triplicate. Additionally, a receiver operating characteristic (ROC) curve was generated to evaluate the diagnostic efficacy of lncRNAs in relation to T2DM.

### Statistical analysis

Statistical analyses and graphs were performed using R 4.1.0, IBM SPSS 26, and GraphPad Prism 9. The independent Student t test was used to determine the statistical significance of differences between two sets of normally distributed data, while the Mann-Whitney U test (i.e., Wilcoxon rank sum test) was used to assess differences between non-normally distributed variables. All *p* values were calculated on a two-sample basis and *p* values < 0.05 were considered statistically significant.

## Results

### Demographic characteristics of participants

The demographic characteristics of the sequencing and the validation participants are shown in Table 1. Blood glucose, glycosylated hemoglobin, urine glucose and triglycerides were all statistically different between the participants who underwent sequencing and those who underwent validation.

**Table 1:** The demographic characteristics of the study population.

	Sequencing(n = 16)				Validation(n = 170)			
	T2DM (n = 8)	CTL (n = 8)	t/z/χ^2^	*P*	T2DM(n = 85)	CTL(n = 85)	t/z/χ^2^	*P*
Age	52.00 ± 7.87	52.13 ± 7.22	0.033	0.974	45.95 ± 13.02	45.87 ± 13.45	0.041	0.968
Gender(Male/Female)	6/2	7/1	–	1.000	57/28	57/28	0.000	1.000
ALT	38.10(30.03，49.25)	34.30(24.85，44.53)	−0.105	0.916	22.10(14.65，35.83)	34.70(20.75，61.70)	−3.679	＜0.001
AST	26.95(18.73，37.08)	30.70(22.43，36.78)	−0.735	0.462	22.55(18.20，28.75)	23.10(15.70，44.95)	−0.683	0.495
ALP	96.66 ± 37.99	82.48 ± 22.47	−0.909	0.379	88.50 ± 25.97	100.10 ± 25.43	−2.652	0.009
TG	1.36(1.01，1.93)	2.48(1.87，3.14)	−2.310	0.021	1.39(0.92，2.11)	2.19(1.30，3.38)	−3.241	0.001
CHOL	5.43 ± 1.75	5.18 ± 1.00	−0.351	0.731	5.38 ± 1.08	5.53 ± 1.55	−0.654	0.514
HDL-C	1.17 ± 0.37	1.18 ± 0.20	0.025	0.980	1.30(1.13，1.40)	1.18 ± 0.41	−3.039	0.002
LDL-C	3.41 ± 1.15	3.02 ± 0.73	−0.807	0.433	3.20 ± 0.70	3.56 ± 0.94	−2.515	0.013
UA	374.63 ± 94.72	402.5 ± 106.37	0.554	0.589	350.37 ± 93.15	306.30(241.90，413.75)	−1.642	0.101
UREA	4.80(4.49，5.29)	4.54(3.27，5.98)	−0.630	0.529	4.77 ± 1.16	5.00(3.87，7.38)	−1.756	0.079
Cr	87.64 ± 18.75	95.71 ± 15.48	0.939	0.364	84.84 ± 12.77	78.06 ± 27.22	1.929	0.057
RBC	5.05 ± 0.63	4.96 ± 0.50	−0.308	0.763	4.78 ± 0.57	4.87 ± 0.67	−0.924	0.357
HGB	154.43 ± 12.15	153.63 ± 11.90	−0.129	0.899	147.08 ± 16.54	152.49 ± 15.74	−2.122	0.035
PLT	193.57 ± 34.86	234.13 ± 35.37	2.230	0.044	242.99 ± 65.87	229.11 ± 63.50	1.359	0.176
WBC	5.37 ± 1.38	5.80 ± 1.30	0.626	0.542	6.88 ± 2.26	6.70(5.55，8.03)	−0.767	0.443
LYMPH%	37.60(23.00，46.90)	33.80(30.05，36.15)	−0.521	0.602	31.35 ± 9.41	29.18 ± 12.02	1.282	0.202
NEUT%	52.64 ± 13.35	54.36 ± 7.69	18.957	＜0.001	59.99 ± 10.78	62.78 ± 12.88	−1.493	0.137
U_KET^*^(N/ P)	7/1	8.0	–	1.000	67/5	36/37	33.706	＜0.001
U_PRO^*^(N/ P)	5/3	8/0	–	0.200	65/7	49/24	11.562	0.001
U_GLU^*^(N/ P)	3/5	8/0	–	0.026	67/5	13/60	82.986	＜0.001
GLU	11.75 ± 5.54	5.45 ± 0.39	−3.214	0.015	5.66 ± 0.87	13.10 ± 6.27	−10.643	＜0.001
HbA1c	9.23 ± 2.71	5.52 ± 0.21	−3.848	0.006	5.45 ± 0.37	10.42 ± 2.37	−15.976	＜0.001

Note: *Data partially missing

### Analysis and screening of differential expression genes

**Fig 1:**
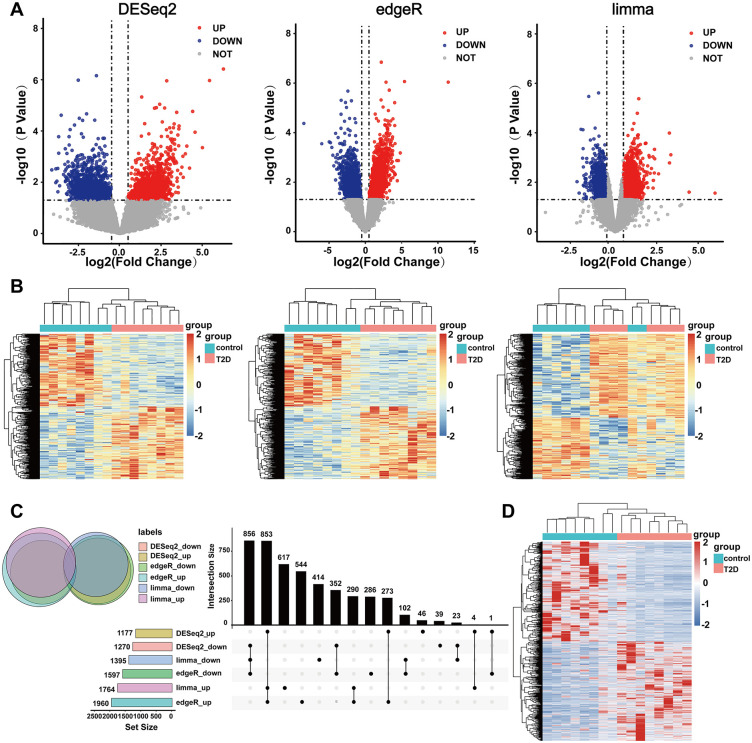
Differentially expressed lncRNAs (DE lncRNAs) in peripheral blood samples between T2DM and healthy control groups. **A.** The volcano plots of DE lncRNAs by three methods (DESeq2, edgeR, and limma). **B.** The heatmaps of DE lncRNAs by three methods (DESeq2, edgeR, and limma). **C.** Venn diagrams and UpSet plots of the 1,709 DE lncRNAs using gene expression profiles. **D.** The heatmap of the overlapped 1,709 DE lncRNAs.

We employed three distinct R packages – limma, DESeq2 and edgeR - to conduct differential analysis on the read counts obtained from high-throughput sequencing, aiming to identify DE lncRNAs between the T2DM and CTL groups. The criteria for screening were established as| log_2_FC | ≥ 1 and *p* < 0.05. Using the limma package, we identified a total of 3,159 statistically significant DE lncRNAs, among these, 1764 were upregulated and 1,395 were downregulated in T2DM samples. The DESeq2 package revealed 2,447 DE lncRNAs, with 1,177 upregulated and 1,270 downregulated in T2DM samples. Furthermore, the edgeR package identified 3,557 DE lncRNAs, consisting of 1,960 upregulated and 1,597 downregulated in T2DM samples (Fig 1A, 1B). Spearman correlation analysis demonstrated strong correlations among the three log_2_FCs (S1 Fig), indicating the robustness of the differential analysis results. To identify the solid DE lncRNAs, we intersected the results of the three methods, and the process along with some of the intermediate results were shown by Venn and UpSet plots (Fig 1C). Finally, a total of 1,709 reliable DE lncRNAs were identified, of which 853 were upregulated and 856 were downregulated compared to CTLs, and the heatmap is shown in Fig 1D.

### Identification of key gene expression modules

**Fig 2:**
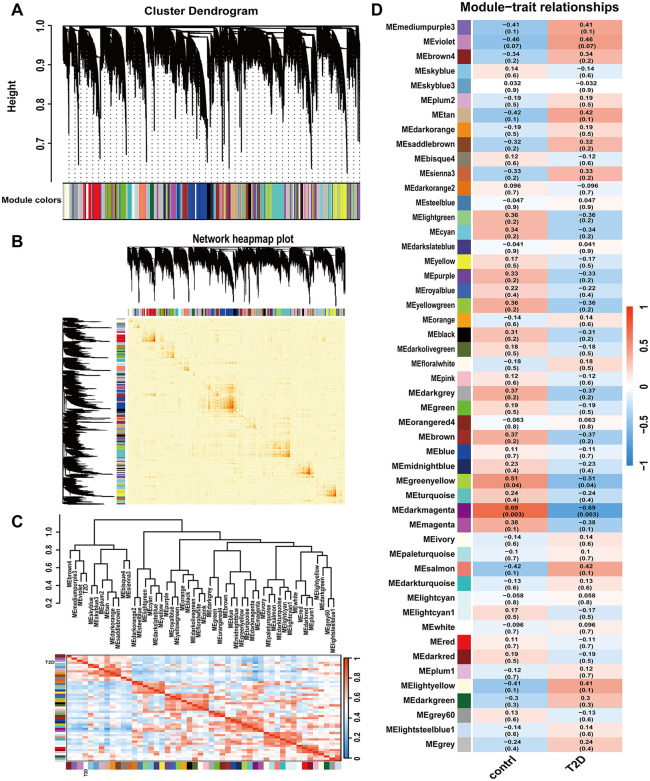
Identification of co-expressed modules and relationship of modules and disease status by WGCNA. **A.** Cluster dendrogram of all genes. **B.** Network heatmap plot of all lncRNAs. **C.** Dendrogram of Module Eigengene (ME) and heatmap of the adjacencies of modules. **D.** Correlation of modules and disease status.

According to the WGCNA package in R, when the soft threshold is set to 9, the scale-free topological fit index reaches 0.84, indicating that the co-expression network conforms to the scale-free network. 50 co-expressed lncRNA modules were clustered in all samples based on the main parameters that minModuleSize = 50 and mergeCutHeight = 0.25. All the modules were marked by colors, and lncRNAs in the grey module were not assigned to any modules (Fig 2A). The primary component of each module, designated as the module eigengene, represents the overall expression levels of lncRNA within that module. To further analyze the relationships among lncRNAs, the topological overlap measure (TOM) was utilised to evaluate their correlations and to construct a TOM matrix. Subsequently, hierarchical clustering analysis was performed based on the dissimilarities among nodes, calculated as (1-TOM) (Fig 2B). Furthermore, analysis of clustering and correlation between modules and T2DM showed that 2 out of 50 modules were significantly associated with T2DM(S2 Table), that is, a total of 1,617 lncRNAs in the greenyellow and darkmagenta modules were negatively correlated with T2DM, which was defined as the key module of this study (Fig 2C, 2D).

### Target gene prediction

By integrating differential expression analysis and WGCNA, we screened a total of 257 lncRNAs that were highly correlated with T2DM with significant changes in expression levels (S2 Fig). Using bioinformatics methods, we predicted 613 target genes for these 257 lncRNAs, including 544 cis-targeted genes and 69 trans-targeted genes (S3 Table).

### Identification and characterization of islet cell classification in an independent scRNA-seq dataset

**Fig 3:**
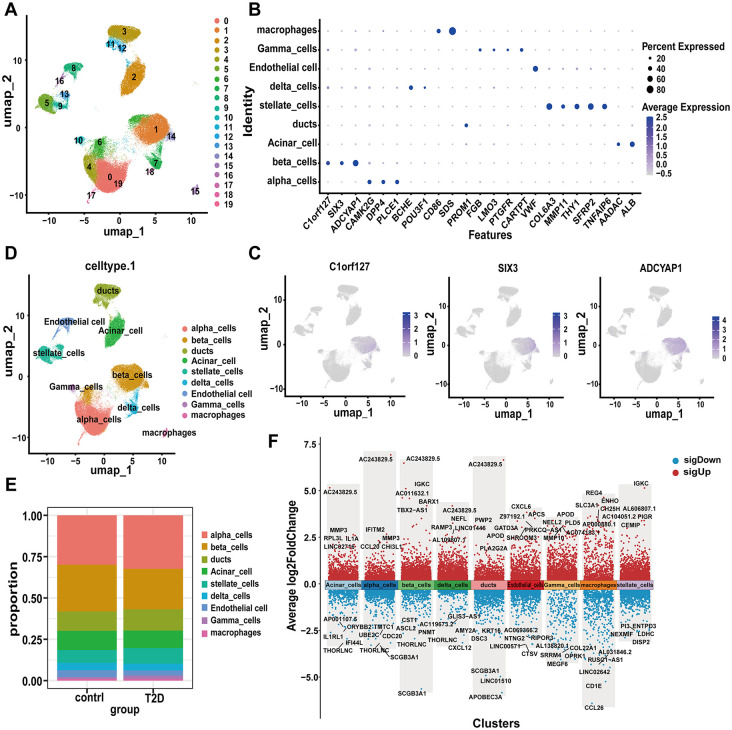
Overview of the single-cell transcriptional profiling of pancreatic cells. **A.** UMAP plot visualization of 20 pancreatic cells clusters. **B.** Dot plot showing the expression of genes that are significantly different among clusters and the percentage of cells expressing these genes in each cluster. **C.** UMAP plots showing expression of classical marker genes from pancreatic β-cells clusters. **D.** UMAP plot visualization of cell-type clusters based on the expression of known marker genes. **E.** Bar plot showing the cell type proportion in different groups. **F.** Differential expression genes of the different cell clusters.

Following stringent quality control and data filtering procedures, 121,194 high-integrity single cells were retained from 17 type 2 diabetes mellitus (T2DM) and 17 controls specimens. The Uniform Manifold Approximation and Projection (UMAP) dimensionality reduction technique revealed 20 transcriptionally distinct cellular clusters, each exhibiting unique transcriptional signatures (Fig 3A). Subsequent cell type classification was conducted through systematic annotation using literature-curated marker genes validated in peer-reviewed studies, with comprehensive annotation results graphically represented in Fig 3B. Distinct expression patterns of canonical β-cell markers within pancreatic β-cell populations were specifically delineated in Fig 3C. Through multi-parameter annotation protocols, we successfully annotated all principal pancreatic lineages (α, β, δ, and γ cells) along with extrapancreatic lineages including ductal epithelium, acinar clusters, stellate cells, vascular endothelia, and macrophage populations (Fig 3D). Quantitative analysis revealed a significant reduction in β-cell compositional distribution within T2DM specimens compared to healthy controls (CTLs) (Fig 3E). To systematically characterize disease-associated transcriptional alterations, we conducted comparative transcriptomic profiling between T2DM and CTL groups across all cellular subtypes. Cell type-specific differentially expressed genes (DEGs) were identified through rigorous Wilcoxon rank-sum testing (*p* < 0.05), with pan-tissue differential expression patterns comprehensively mapped in Fig 3F. Given the central pathophysiological role of pancreatic β-cells in the pathogenesis of T2DM, 4,693 β-cell-specific DEGs are listed in S4 Table.

### LncRNA-mRNA Co-expression networks and functional enrichment analysis

**Fig 4:**
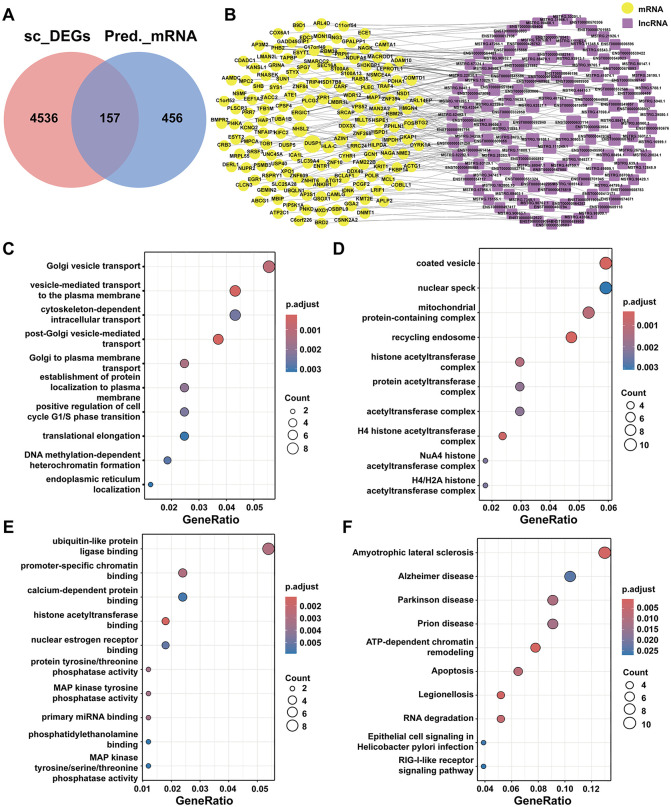
LncRNA-mRNA Co-expression networks and functional enrichment analysis. **A.** Venn diagram showing 157 DEGs. B. lncRNA-mRNA co-expression network. **C.** GO terms of biological process (BP). **D.** GO terms of cellular component (CC). **E.** GO terms of molecular function (MF). **F.** KEGG pathways.

A total of 157 differentially expressed mRNAs were acquired by intersecting the 4693 differentially expressed mRNAs in pancreatic β-cells with 613 target gene results predicted by the screened differentially expressed lncRNAs (Fig 4A). As a single lncRNA can regulate multiple target mRNAs, we ultimately obtained 135 corresponding differentially Expressed lncRNAs, and the lncRNA-mRNA co-expression network is depicted in Fig 4B. To further explore the possible mechanisms involved in lncRNA-mRNA, GO and KEGG enrichment analyses were performed on these differentially expressed mRNAs. GO pathway analysis illustrated that DEGs were associated with Golgi vesicle transport, vesicle-mediated transport to the plasma membrane (BP), coated vesicle, nuclear speck (CC), and ubiquitin-like protein ligase binding, promoter-specific chromatin binding (MF) (Fig 4C-4E). KEGG analysis suggested that DEGs were mainly involved in amyotrophic lateral sclerosis, Alzheimer’s disease, Parkinson’s disease, prion disease, ATP-dependent chromatin remodeling, and apoptosis, among others (Fig 4F).

### Identification and validation of diagnostic biomarkers for T2DM

**Fig 5:**
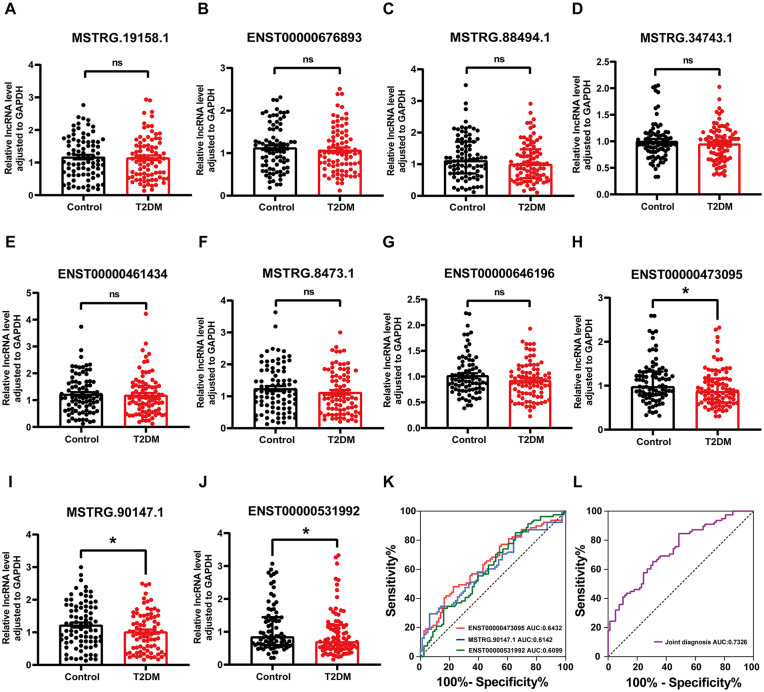
Identification and validation of diagnostic biomarkers for T2DM. A-J. Comparison of peripheral blood lncRNA expression levels between T2DM and healthy controls via RT-qPCR. The relative expression levels were expressed as 2^−ΔΔCt^. **K.** ROC curve of ENST00000473095, MSTRG90147.1and ENST00000531992. **L.** ROC curve of the combined diagnostic performance of ENST00000473095, MSTRG90147.1 and ENST00000531992. *: *p* ＜ 0.05.

Following the identification of differentially expressed DE lncRNAs between individuals with T2DM and healthy controls, we integrated the results from three screening methods and selected the top 10 candidate lncRNAs for further validation via qRT-PCR (S5 Table). This validation was performed in an expanded cohort comprising 85 T2DM patients and 85 matched healthy controls. Quantitative analysis revealed that ENST00000473095, MSTRG90147.1and ENST00000531992 were significant downregulation in T2DM patients compared to the endogenous control group (p < 0.05), with ENST00000473095 being consistent with the sequencing results, while MSTRG90147.1 and ENST00000531992 were opposite to the sequencing results (Fig 5A-5J). This suggests that ENST00000473095, MSTRG90147.1 and ENST00000531992 may play a functional role in T2DM pathogenesis and could serve as a potential biomarker for the disease. Furthermore, receiver operating characteristic (ROC) curve analysis revealed that ENST00000473095 exhibited an area under the curve (AUC) of 0.6432 (95%CI: 0.5573–0.7291), the AUC of MSTRG90147.1 was 0.6142 (95%CI: 0.5258–0.7205), and the AUC of ENST00000531992 was 0.6099 (95%CI: 0.5233–0.6965) (Fig 5K). The AUC of the combined score of these three lncRNAs is 0.7326 (95% CI: 0.6554–0.8098) (Fig 5L), which exceeds the AUCs of the individual lncRNAs (0.61–0.64), confirming the advantage of combining these lncRNAs as biomarkers.These findings support the potential utility of ENST00000473095, MSTRG90147.1 and ENST00000531992 as a biomarker for distinguishing T2DM patients from healthy individuals, highlighting its diagnostic relevance.

## Discussion

Type 2 diabetes mellitus (T2DM) represents a complex metabolic disorder characterized by progressive β-cell dysfunction and insulin resistance, although the precise molecular triggers initiating these pathological processes remain incompletely understood. Recent breakthroughs in multi-omics approaches, particularly scRNA-seq, coupled with biorthogonal molecular tracking techniques, have fundamentally transformed our capacity to delineate early disease signatures across multiple tissue compartments.

In this study, we systematically identified blood-pancreas shared lncRNAs as potential systemic biomarkers in treatment-naïve patients with new-onset T2DM, through integrative analysis of peripheral blood transcriptome profiles and pancreatic islet scRNA-seq data. Our findings establish a critical connection between peripheral blood biomarkers and tissue-specific disease mechanisms, providing both novel insights into early T2DM pathogenesis and promising avenues for diagnostic innovation.

Our comprehensive analysis revealed 1709 differentially expressed lncRNAs in peripheral blood, of which 257 were identified as high-priority candidate through weighted gene co-expression network analysis (WGCNA). This extensive dysregulation pattern strongly supports the systemic nature of T2DM pathogenesis. Particularly compelling is the observed co-expression network between blood-derived lncRNAs and β-cell-specific mRNAs, suggesting the existence of a conserved regulatory axis connecting the circulatory system and pancreatic islets. These findings corroborate emerging evidence that lncRNAs serve as critical mediators of inter-organ communication in metabolic disorders. Two bootable examples include: βLinc1, which preserves β-cell identity by regulating PDX1-dependent transcriptional programs [12]. LINK-A, which orchestrates adipose-liver metabolic crosstalk via AKT signaling modulation [13]. Our study significantly advances this field by demonstrating that blood-derived lncRNAs can reflect pancreatic transcriptional stress during the pre-hyperglycemic phase. This discovery positions these molecules as potential early-warning biomarkers for emerging β-cell dysfunction, offering a critical window for therapeutic intervention before irreversible damage occurs.

Functional enrichment analysis of co-expressed mRNAs identified three key pathways implicated in T2DM progression: focal adhesion, chromatin remodeling and neurodegenerative pathways. Each pathway offers distinct yet complementary insights into disease mechanisms. Focal adhesion dynamics are essential for insulin granule trafficking exocytosis and mediated β-cell survival under metabolic stress conditions [14]. Focal adhesion dynamics may represent a compensatory mechanism during early β-cell dysfunction. Chromatin remodeling regulates β-cell plasticity and identity maintenance and facilitates expansion during metabolic compensation [15]. Chromatin remodeling is potential epigenetic driver of β-cell failure in prolonged hyperglycemia. Neurodegenerative pathway reveals unexpected molecular convergence between T2DM and neurodegeneration. Mitochondrial dysfunction and oxidative stress emerge as shared mechanisms [16–18]. Mitochondrial lncRNAs may coordinately regulate oxidative phosphorylation efficiency [19], β-cell functional integrity and neuronal homeostasis [20]. These findings position specific lncRNAs (e.g., ENST00000473095) as potential molecular nodes in metabolic-neuronal crosstalk.

In our analysis of target gene prediction, we identified both cis- and trans-regulatory relationships for 257 high-priority lncRNAs, resulting in 544 cis-target genes and 69 trans-target genes. Most predicted interactions were cis-regulatory, suggesting that chromatin regions near lncRNAs regulate adjacent genes. However, a smaller number of trans-target genes were also identified, indicating potential long-range or network-based regulatory mechanisms, such as interactions mediated through chromatin looping or transcriptional complexes. Notably, cis-target genes were enriched in pathways related to chromatin organization and insulin secretion, emphasizing their importance for β-cell function. For example, ENST00000473095 was predicted to cis-regulate PDX1, a key regulator of β-cell identity and function. This finding aligns with earlier research linking lncRNAs to β-cell transcriptional networks [12]. Interventions targeting this regulatory axis, such as antisense oligonucleotides (ASOs) or small-molecule inhibitors, could prove effective in preserving β-cell mass during the prediabetic stage. Similar to research on cardiovascular biomarkers like NT-proBNP, longitudinal studies tracking the dynamic changes of lncRNA from normoglycemia to T2DM, are critical for validating their predictive value [21]. Furthermore, integrating multi-omics data can uncover synergistic biological pathways. Recent studies have successfully linked HNF1A-driven β-cell heterogeneity to mitochondrial dysfunction, serving as prime examples of such integrative research [9,22,23].Although there were fewer trans-targets, they included genes related to neurodegenerative and metabolic pathways, indicating potential broader systemic influences.

The regulatory relationships we identified played a crucial role in our strategy for prioritizing biomarkers. We focused on lncRNAs whose target genes were not only differentially expressed in pancreatic β-cells but also functionally involved in the development of T2DM. By integrating predictions from both cis- and trans-regulatory interactions with co-expression networks, we were able to prioritize specific lncRNAs, including ENST00000473095, MSTRG.90147.1, and ENST00000531992. These lncRNAs were significantly dysregulated and plausibly linked to β-cell dysfunction. Although we could not experimentally validate all the predicted interactions in this study, our computational approach provided a sound rationale for selecting biomarkers that had functional significance beyond just changes in expression levels. Future studies using CRISPR-based perturbation techniques or dual-luciferase assays could validate these cis/trans-interactions and could clarify their roles in T2DM progression.

The validation of ENST00000473095, MSTRG.90147.1and ENST00000531992 as diagnostic biomarkers, despite moderate AUC values (0.64, 0.61and 0.61), reflects challenges inherent to single-marker approaches. The combined AUC of the three lncRNAs exceeds that of a single lncRNA, indicating that their combination as biomarkers significantly improves diagnostic accuracy. This improvement enhances the potential clinical relevance of our findings. Recent studies emphasize combinatorial biomarker panels to enhance specificity. Integrating lncRNAs with protein markers or epigenetic modifications could refine T2DM diagnostics [24,25]. Furthermore, nanoparticle delivery systems offer promising avenues for therapeutic lncRNA modulation, as demonstrated in preclinical models of obesity-associated insulin resistance [26,27].

Our multi-modal approach, integrating bulk transcriptomics, scRNA-seq, and machine learning algorithms, provides a comprehensive framework for T2DM biomarker discovery. However, reliance on public scRNA-seq data introduces potential batch effects, a limitation noted in recent single-cell meta-analyses [28,29]. Additionally, while WGCNA identified co-expression modules linked to β-cell stress, functional validation remains imperative. Techniques such as CRISPR interference paired with spatial transcriptomics could elucidate lncRNA localization and interaction networks in human islets [30]. The modest sample size in the discovery cohort (n = 8/group) also limits generalizability, necessitating replication in larger, ethnically diverse cohorts.

Through the application of state-of-the-art sequencing technologies and cross-tissue integration strategies, this research tentatively suggests that blood-derived lncRNAs serve as early biomarkers reflecting β-cell stress and systemic dysregulation, and these lncRNAs may bridge peripheral blood biomarkers with tissue-specific pathophysiology in T2DM. Although substantial hurdles persist in translating these findings into clinical practice, they highlight the significance of discovering systemic biomarkers to decipher the intricate nature of metabolic diseases. Looking ahead, future research endeavors should prioritize functional validation of these lncRNAs, the development of combinatorial diagnostic approaches, and innovation in therapeutic strategies. By doing so, we can hope to arrest the progression of T2DM at its incipient stages, ultimately improving patient outcomes and public health.

## Supporting information

S1 Fig The correlation analysis between the log_2_FCs calculted by three R packages. (DOCX)

S2 Fig Venn diagram showing intersecting feature lncRNAs in T2DM selected by differential expression analysis and WGCNA. (PDF)

S1 Table PCR primers used for validation of profiling results. (DOCX)

S2 Table LncRNAs contained in the greenyellow and the darkmagenta module. (XLSX)

S3 Table Cis trans target. (XLSX)

S4 Table Differentially expressed genes in pancreatic beta cells. (XLSX)

S5 Table The top 10 differentially expressed LncRNAs between T2DM and control groups. (DOCX)
