# Supplementary figures and images for: Integrated transcriptome and single-cell sequencing analysis identify blood-pancreas shared lncRNA biomarkers in new-onset T2DM
Source: PLoS One. 2026 Mar 31;21(3):e0345359. doi: 10.1371/journal.pone.0345359 (PMC13037964; doi:10.1371/journal.pone.0345359)

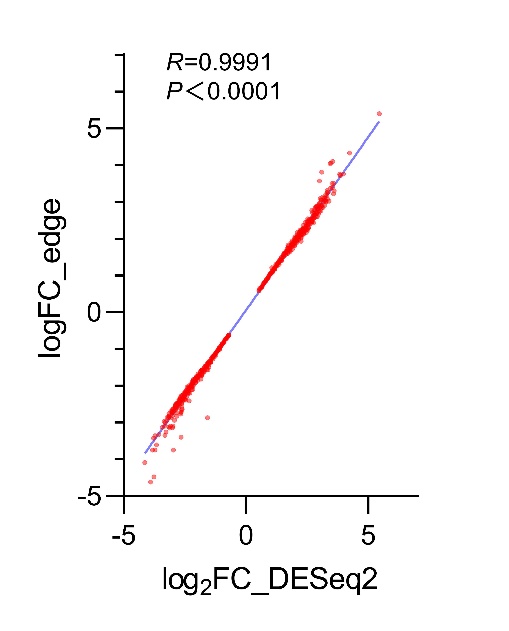

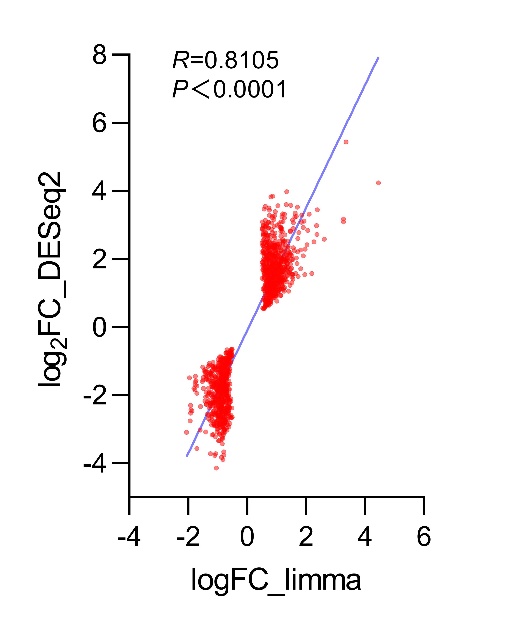

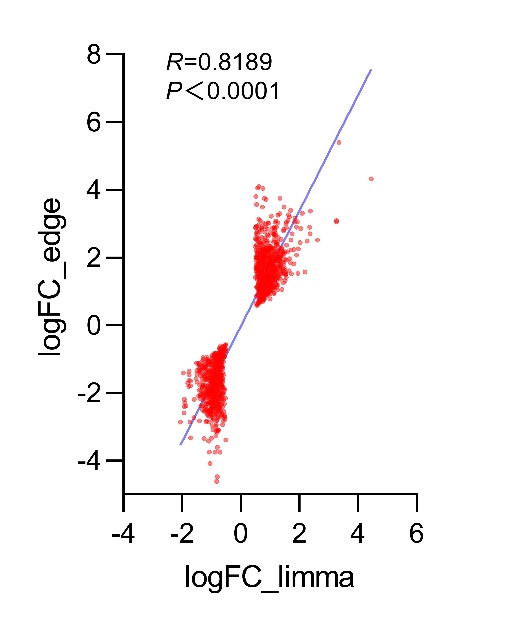


S1 Fig. The correlation analysis between the log2FCs calculted by three R packages.

Supplement: S1 Fig — (DOCX) [file pone.0345359.s001.docx]
