# Supplementary material for: Integrated transcriptome and single-cell sequencing analysis identify blood-pancreas shared lncRNA biomarkers in new-onset T2DM
Source: PLoS One. 2026 Mar 31;21(3):e0345359. doi: 10.1371/journal.pone.0345359 (PMC13037964; doi:10.1371/journal.pone.0345359)
Supplement: S2 Fig — (PDF) [file pone.0345359.s002.pdf]

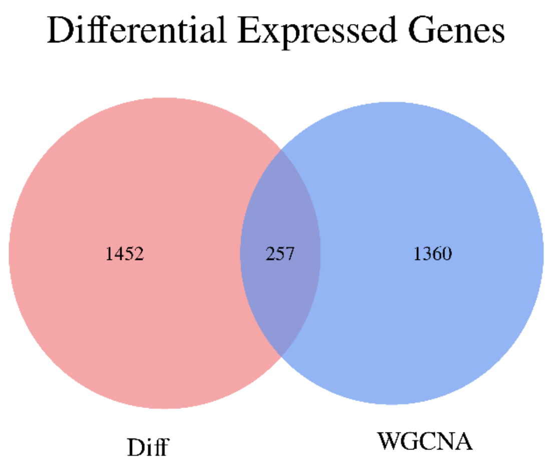

S2 Fig. Venn diagram showing intersecting feature lncRNAs in T2DM selected by differential expression analysis and WGCNA.
