# Supplementary material for: Integrated transcriptome and single-cell sequencing analysis identify blood-pancreas shared lncRNA biomarkers in new-onset T2DM
Source: PLoS One. 2026 Mar 31;21(3):e0345359. doi: 10.1371/journal.pone.0345359 (PMC13037964; doi:10.1371/journal.pone.0345359)
Supplement: S1 Table — (DOCX) [file pone.0345359.s003.docx]

**S1 Table. PCR primers used for validation of profiling results.**

| Transcript | Forward Primer (5'→3') | Reverse Primer(5'→3') |
| --- | --- | --- |
| GAPDH | ACACCCACTCCTCCACCTTTG | TCCACCACCCTGTTGCTGTAG |
| MSTRG.90147.1 | CTTTGTAGCAAGTCCAGAGGTCCTG | GGCACTAAACAAATTGGTGGCACTG |
| ENST00000676893 | AAACAAGTGGGTGAGTCGCAAGAG | GGGCTGTGGTGGGAGAGAGTC |
| ENST00000473095 | ACATCACTTACCAGGAGCCTCAGG | GTCGGCACTGTTTCAGCACCTC |
| ENST00000531992 | GCGGAGGTGAAGGGCTTTGTC | CAAGGTTCCAGGGGTGCTTTCG |
| MSTRG.88494.1 | GGGTCACTGGAGGCTGGCTAC | TGGGAAGGCTCTGGGTCTCTTTG |
| MSTRG.34743.1 | GACGCCCGACCCTCCTCTTC | ACAACTAACACACGCAGACTACAGG |
| ENST00000461434 | ATTCAAAGACCCGCAGCAACCC | ACAACCACCCACCTACTGAGTCC |
| MSTRG.92024.1 | AGACCAGCCTGAGTGATGTAGTGAG | TAAGCAATCCTCCCACCTCATCCC |
| ENST00000646196 | GCAGCCGTGGTGACAGAAGTG | AGAGTTCCTGTTCCAAGCGTTCAC |
| MSTRG.8473.1 | GCTCCCTATAGCCAACTTGATCACC | ACTAACGCTGTCTGGATCTGGTCTC |
| MSTRG.19158.1 | ATCCCAGCGCAAACACTGACAG | TGGTCTCGAACTCTCAACCTCAGG |
