# Supplementary material for: Integrated transcriptome and single-cell sequencing analysis identify blood-pancreas shared lncRNA biomarkers in new-onset T2DM
Source: PLoS One. 2026 Mar 31;21(3):e0345359. doi: 10.1371/journal.pone.0345359 (PMC13037964; doi:10.1371/journal.pone.0345359)
Supplement: S5 Table — (DOCX) [file pone.0345359.s007.docx]

**S5 Table. The top 10 differentially expressed LncRNAs between T2DM and control groups.**

| Transcription ID | DESeq2 | | edgeR | | | limma | | Regulation | chr |
| --- | --- | --- | --- | --- | --- | --- | --- | --- | --- |
|  | log_2_FC | *P* | logFC | *P* | logFC | | *P* |  |  |
| MSTRG.90147.1 | 3.178 | 0.0002 | 3.096 | 0.0005 | | 3.266 | 0.0001 | Up | chr6：26554122-26554475 |
| ENST00000676893 | 2.609 | 0.0060 | 2.513 | 0.0042 | | 1.718 | 0.0074 | Up | chr12：6966926-6970780 |
| ENST00000531992 | 2.269 | 0.0005 | 2.241 | 0.0003 | | 1.817 | 0.0003 | Up | chr8：143991294-143992078 |
| ENST00000473095 | -2.332 | 0.0001 | -2.190 | 0.0001 | | -1.578 | 0.0110 | Down | chr7：56102636-56106494 |
| MSTRG.34743.1 | 2.125 | 0.0050 | 2.096 | 0.0033 | | 1.327 | 0.0122 | Up | chr14：52730132-52731360 |
| MSTRG.88494.1 | -2.269 | 0.0009 | -2.064 | 0.0004 | | -1.376 | 0.0026 | Down | chr5：172830161-172831852 |
| ENST00000461434 | 2.038 | 0.0011 | 1.992 | 0.0005 | | 1.356 | 0.0010 | Up | chr1：151254758-151263076 |
| MSTRG.8473.1 | 1.988 | 0.0061 | 1.939 | 0.0031 | | 1.507 | 0.0017 | Up | chr1：85672141-85672498 |
| MSTRG.19158.1 | 1.985 | 0.0031 | 1.934 | 0.0013 | | 1.552 | 0.0006 | Up | chr10：121903195-121903491 |
| ENST00000646196 | -2.020 | ＜0.0001 | -1.915 | ＜0.0001 | | -1.343 | 0.0011 | Down | X：41334162-41350274 |
